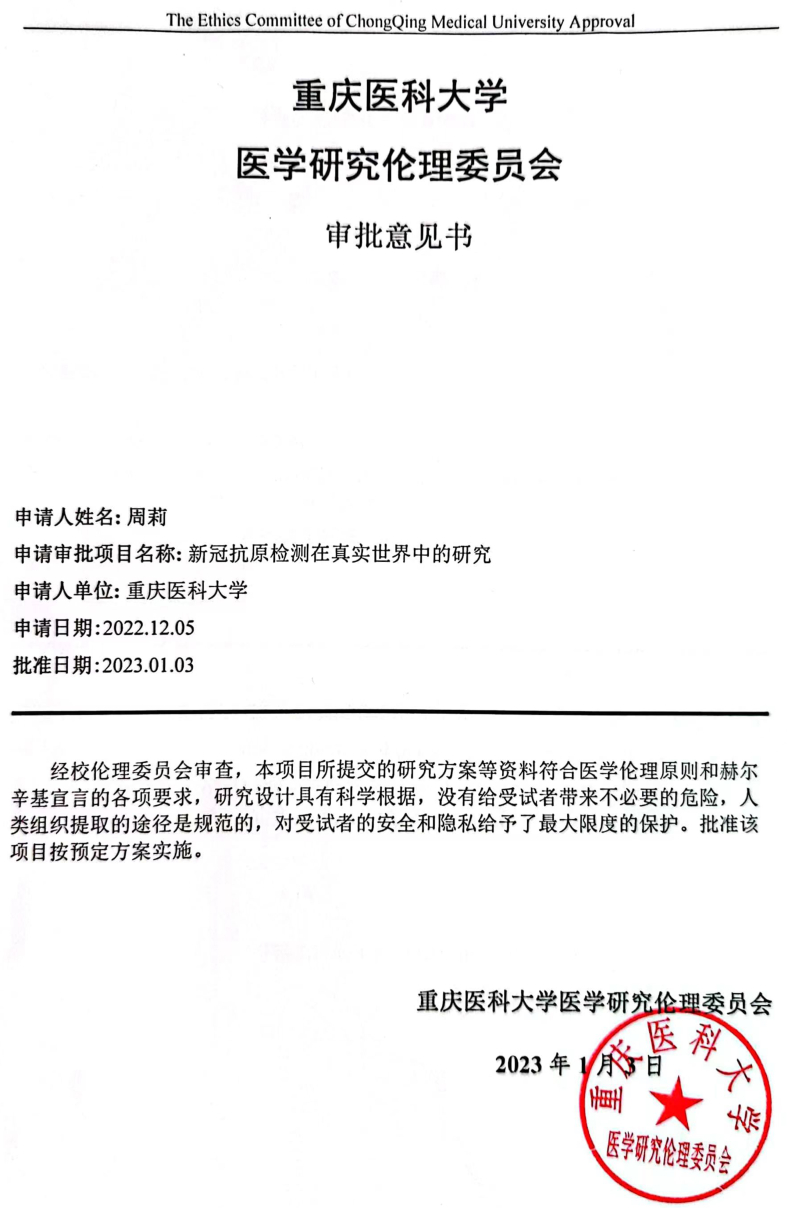# Efficacy of rapid antigen self-testing for SARS-CoV-2 screening: Real-world evidence from a prospective cohort study

**DOI:** 10.1016/j.gendis.2023.101151

**Published:** 2023-10-28

**Authors:** Yaoyue Hu, Bin Peng, Jie Fan, Zhu Yang, Jianjiang Xue, Quanxin Long, Jie Xia, Yuan Hu, Xuefei Cai, Li Zhou, Ailong Huang

**Affiliations:** a School of Public Health, Chongqing Medical University, Chongqing 400016, China; b The Second Affiliated Hospital of Chongqing Medical University, Chongqing 400016, China; c Clinical Laboratory, University-Town Hospital of Chongqing Medical University, Chongqing 401331, China; d Key Laboratory of Molecular Biology on Infectious Diseases Designated by the Chinese Ministry of Education, Chongqing Medical University, Chongqing 400016, China

Antigen rapid diagnostic tests (Ag-RDTs) have been considered and implemented as an important diagnostic and screening tool to identify SARS-CoV-2 infections in community settings.^1^ Ag-RDTs are less sensitive, particularly in asymptomatic populations, compared with laboratory-based viral nucleic acid amplification tests (NAATs) such as reverse transcription polymerase chain reaction (RT-PCR).^2^ However, taking into account the facts that Ag-RDTs are effective for identifying most contagious individuals, they are faster and less expensive than RT-PCR, as well as that RT-PCR could produce positive results for weeks to months after the infection,^2^ WHO recommends Ag-RDTs be offered as COVID-19 self-testing for screening purposes in addition to professionally administered testing services regardless of the community transmission level.^1^ Although Ag-RDTs have been utilized in mass screening in Slovakia and Liverpool,^2^ there is a dearth of real-world evidence from prospective cohort studies on the efficacy of Ag-RDT self-testing in comparison to RT-PCR due to the challenges in performing both testing consecutively at the same time over the follow-up. The study aims to evaluate the effectiveness of rapid antigen self-testing for screening SARS-CoV-2.

The strict zero-COVID policy in China was loosened on December 7, 2022, when the new guidelines on COVID-19 were issued, after which SARS-CoV-2 infections surged. With no mass RT-CPR testing being organized by the government anymore, it is important to evaluate Ag-RDTs as an alternative screening tool in the application with real-world data. During the COVID-19 outbreak, 17,655 residents of the Chongqing Medical University campus were isolated and subjected to Ag-RDT self-testing (M&D Covid-19 Antigen Rapid Test, Chongqing M&D Biotechnology, Chongqing, China) three times a day and daily RT-PCR testing (COVID-19 Multiplex RT-PCR Kit, Shengxiang Biotechnology, China, registration no. 20203400064) between November 11 and November 28, 2022. A total of 26 (0.15%) were infected and tested positive by both Ag-RDT and RT-PCR; the rest consistently tested negative by both Ag-RDT and RT-PCR. The figure depicts the timeline of testing positive by Ag-RDT, sample collection for RT-PCR, and receiving positive RT-PCR results in COVID-19 cases. The mean turnaround time for RT-PCR was 13.39 h (standard deviation/SD: 4.74). For cases whose RT-PCR sample collection occurred before Ag-RDT (*n* = 20), the mean time between sample collection of these two tests was 15.39 h (SD: 5.15); it was 5.35 h if RT-PCR sample collection occurred after Ag-RDT (*n* = 6). Among the 26 cases, 16 (61.5%) had positive RT-PCR results on average 3.81 h (SD: 3.33) before testing positive by Ag-RDT; four had positive Ag-RDT results while awaiting their RT-PCR results after having their sample collected, while six cases (23.1%) had positive Ag-RDT results before positive RT-PCR results. The mean time for having positive Ag-RDT results prior to positive RT-PCR results was 13.32 h (SD: 8.35).

The positive agreement between Ag-RDT and TR-PCR was 82.4% (14 out of 17, 95% CI: 56.6%–96.2%) in a 6-h time interval between receiving the results of RT-PCR and Ag-RDT, 100.0% (23 out of 23, 95% CI: 85.2%–100.0%) in a 12-h time interval, and 100.0% (25 out of 25, 95% CI: 86.3%–100.0%) in a 24-h time interval. There was a trend for the cycle thresholds (Ct) value to decrease with increasing time intervals (Pearson correlation coefficient: N gene = −0.413, *P* = 0.0448; ORF1lab gene = −0.466, *P* = 0.0216), suggesting a dose-response relationship in which the lower the Ct value, the earlier the Ag-RDT was positive ahead of RT-PCR.

While it is difficult to determine the false-positive rate of Ag-RDTs in mass testing due to the lack of confirmatory RT-PCR results, with such data available we found no false positive of the Ag-RDT. Our observation that 10 out of 26 cases tested positive by Ag-RDT prior to obtaining their RT-PCR results suggests that the potential for using Ag-RDTs when viral prevalence is relatively high could be much more promising than previously reported. The city-wide rapid antigen testing pilot in Liverpool, UK revealed that about 10% of persons with high viral loads were missed.^3^ We found no false negative of the Ag-RDT as all cases confirmed by RT-PCR also tested positive by Ag-RDT. Since false negative results of Ag-RDTs could occur in the early stage of the infection when the viral load is low, García-Fiñana et al^4^ proposed that serial antigen testing for those people could improve sensitivity. This approach was used in our investigation which might have helped to lower the possibility of false negative. Although Ag-RDTs are less sensitive than RT-PCR, the better sensitivity of Ag-RDTs in samples with lower Ct values^3^ was also evidenced in our investigation.

Screening is central to helping the societies to re-open and contain the SARS-CoV-2 transmission.^5^ In order to efficiently reduce viral transmission in the population, testing frequency and turnaround time are as crucial as test sensitivity.^5^ Although the hospital gave our samples priority for testing, it still took more than 13 h on average to receive the RT-PCR result. In our investigation, 38.5% of cases were aware of the infection based on their antigen test results at least 6 h before RT-PCR confirmation. Since we found that the Ag-RDT performed as well as RT-PCR to identify infections, coupled with Ag-RDTs being quick and inexpensive, Ag-RDT self-testing can ease the excessive demand for and heavy dependence on RT-PCR testing, thus reducing the economic and societal costs of mass testing.

The use of Ag-RDTs in conjunction with RT-PCR allowed us to identify infected persons and isolate their close contacts early, thus decreasing the secondary attack rate among close contacts residing on the university campus (11 infected out of 57 close contacts, 19.3%), as compared with their counterparts living in Yuzhong District of Chongqing Municipality where the university is located (1225 infected out of 2286 close contacts, 53.6%). Ag-RDT self-testing at regular intervals therefore could help to flatten the curve of new infections, preventing the overflow of COVID-19 patients into hospitals and the collapse of the healthcare system. With new infections on the rise after the lifting of restrictions, the latter is particularly important in China, where there are large urban-rural disparities in the availability and quality of healthcare Figure 1.

**Figure 1:**
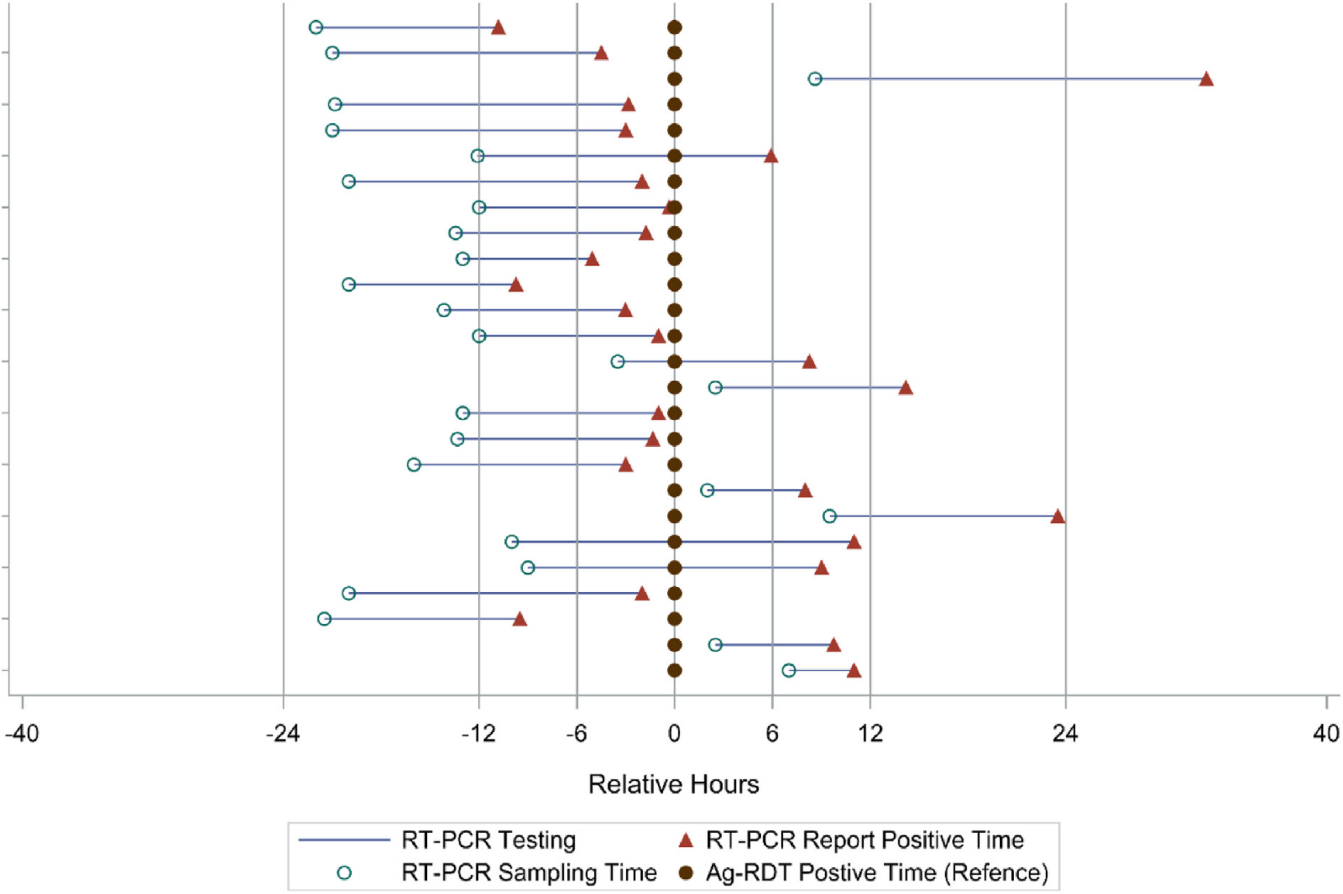
Time of testing positive by Ag-RDT and RT-PCR among COVID-19 cases.

## Author contributions

Yaoyue Hu and Li Zhou wrote the original manuscript and were involved in the investigation. Li Zhou acquired the data. Bin Peng performed the data analysis. Ailong Huang conceived and designed the study. All authors had access to the data, revised the manuscript, and approved the final version.

## Conflict of interests

Ailong Huang is one of the Editors-in-Chief and a member of *Genes & Diseases* Editorial Board. To minimize bias, he was excluded from all editorial decision-making related to the acceptance of this article for publication. The remaining authors declare no conflict of interest.

## Funding

This work is supported by the State Laboratory of Respiratory Diseases, Guangzhou, China (No. TL22-15) and the Municipal Natural Science Foundation of Chongqing, China (No. stc2020jscx-fyzxX0003) and Chongqing Biomedical R&D Major Special Project (China) (No. CSTB2022TIAD-STX0013).

## Data availability

The data are available on request from the authors.

## Figures and Tables

**figs1:**
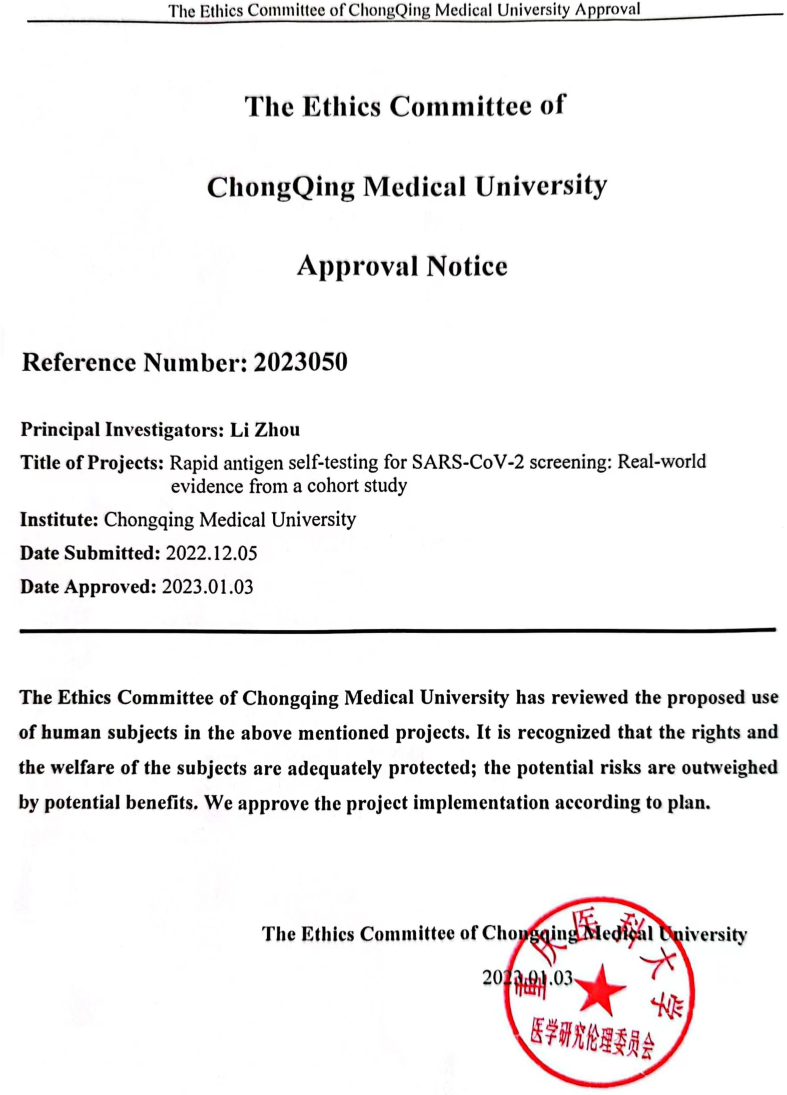


**figs2:**