# Supplementary material for: Efficacy of rapid antigen self-testing for SARS-CoV-2 screening: Real-world evidence from a prospective cohort study
Source: Genes Dis. 2023 Oct 28;11(5):101151. doi: 10.1016/j.gendis.2023.101151 (PMC11176647; doi:10.1016/j.gendis.2023.101151)
Supplement: Multimedia component 1 [file mmc1.docx]

**2.Methods**

***2.1 Study sample***

During the COVID-19 outbreak in Chongqing Municipality, 17,655 residents of the Chongqing Medical University campus had Ag-RDT and RT-PCR tests in the isolation period between November 11 and November 28, 2022. In this prospective cohort study, all study subjects were not infected by SARS-CoV-2 on November 11, 2022. During the 18 days of follow-up, Ag-RDT self-testing was carried out three times a day with a time interval of 4-6 hours, whereas RT-PCR test was performed once a day. Once study subjects tested positive by RT-PCR, they were transferred to designated hospitals for treatment; while their close contacts were quarantined in a separate place where their health status was monitored. Demographic characteristics and laboratory-based RT-PCR results were extracted from electronic medical records. This study was approved by the Ethical Review Committee at Chongqing Medical University.

***2.2 Ag-RDT***

The Ag-RDT used in this study was the M&D Covid-19 Antigen Rapid Test (Chongqing M&D Biotechnology, Chongqing, China). The test was conducted by the study subjects and the results were reported online via a smart phone application developed by Chongqing Medical University.

***2.3 RT-PCR***

The RT-PCR test was performed using the COVID-19 Multiplex RT-PCR Kit (Shengxiang Biotechnology, China, registration no. 20203400064) by medical personnel at the University-Town Hospital of Chongqing Medical University in accordance with the manufacturer’s instructions. The entire process was controlled through isolation and amplification of the synthetic internal control. Cycle thresholds (Ct) for ORF1ab and N genes were recorded.

***2.4 Statistics***

Sample characteristics were described with number (%) and mean (standard deviation, SD). Ag-RDT positive rate and positive agreement between the Ag-RDT and RT-PCR with 95% Clopper-Pearson confidence interval (CI) were calculated. The association between the Ct value and the time interval between the RT-PCR and Ag-RDT results (i.e. the time receiving the RT-PCR result subtracted from the time obtaining the Ag-RDT result) was analyzed using linear regression and Pearson correlation coefficient. All data analyses were performed using SAS 9.4 (SAS Institute Inc, USA) with *p*<0.05 considered as statistically significant.
